# Functional analysis of structurally related soybean GmWRKY58 and GmWRKY76 in plant growth and development

**DOI:** 10.1093/jxb/erw252

**Published:** 2016-06-21

**Authors:** Yan Yang, Yingjun Chi, Ze Wang, Yuan Zhou, Baofang Fan, Zhixiang Chen

**Affiliations:** ^1^Department of Horticulture, Zijingang Campus, 866 Yuhangtang Road, Zhejiang University, Hangzhou 310058, China; ^2^Department of Botany and Plant Pathology, 915W. State Street, Purdue University, West Lafayette, IN 47907, USA

**Keywords:** AtWRKY54, AtWRKY70, flowering, plant growth, soybean, virus-induced gene silencing, WRKY transcription factors.

## Abstract

WRKY transcription factors constitute a large protein superfamily with a predominant role in plant stress responses. In this study we report that two structurally related soybean WRKY proteins, GmWRKY58 and GmWRKY76, play a critical role in plant growth and flowering. GmWRKY58 and GmWRKY76 are both Group III WRKY proteins with a C2HC zinc finger domain and are close homologs of AtWRKY70 and AtWRKY54, two well-characterized Arabidopsis WRKY proteins with an important role in plant responses to biotic and abiotic stresses. GmWRKY58 and GmWRKY76 are both localized to the nucleus, recognize the TTGACC W-box sequence with a high specificity, and function as transcriptional activators in both yeast and plant cells. Expression of *GmWRKY58* and *GmWRKY76* was detected at low levels in roots, stem, leaves, flowers, and pods. Expression of the two genes in leaves increased substantially during the first 4 weeks after germination but steadily declined thereafter with increased age. To determine their biological functions, transgenic Arabidopsis plants were generated overexpressing *GmWRKY58* or *GmWRKY76*. Unlike *AtWRKY70* and *AtWRKY54*, overexpression of *GmWRKY58* or *GmWRKY76* had no effect on disease resistance and only small effects on abiotic stress tolerance of the transgenic plants. Significantly, transgenic Arabidopsis plants overexpressing *GmWRKY58* or *GmWRKY76* flowered substantially earlier than control plants and this early flowering phenotype was associated with increased expression of several flowering-promoting genes, some of which are enriched in W-box sequences in their promoters recognized by GmWRKY58 and GmWRKY76. In addition, virus-induced silencing of *GmWRKY58* and *GmWRKY76* in soybean resulted in stunted plants with reduced leaf expansion and terminated stem growth. These results provide strong evidence for functional divergence among close structural homologs of WRKY proteins from different plant species.

## Highlight

A functional analysis is conducted on two structurally related soybean WRKY proteins, GmWRKY58 and GmWRKY76, and they are found to play a critical role in plant growth and flowering.

## Introduction

WRKY proteins, first discovered just over 20 years ago, are a class of sequence-specific DNA-binding transcription factors (Rushton *et al.*, 2010). Although genes encoding WRKY proteins are found in non-photosynthetic eukaryotes, it is in higher plants where genes encoding WRKY proteins have greatly proliferated into large families, with more than 70 members in *Arabidopsis thaliana* and more than 100 members in rice (Wu *et al.*, 2005; Zhang and Wang, 2005; Rushton *et al.*, 2010). WRKY proteins contain the highly conserved WRKY domain, with the almost invariant WRKYGQK sequence at the N-terminus followed by a C2H2 or C2HC zinc-finger motif (Rushton *et al.*, 2010). WRKY proteins were initially classified into three groups based on the number and sequence of the conserved WRKY zinc-finger motifs (Eulgem *et al.*, 2000). Group I WRKY proteins contain two C2H2 zinc-finger motifs, whereas Group II WRKY proteins contain one C2H2 zinc-finger motif, and Group III WRKY proteins contain one C2HC zinc-finger motif. Further analyses have shown that Group II WRKY proteins can be further divided into five subgroups (IIa, IIb, IIc, IId, and IIe) (Zhang and Wang, 2005; Rushton *et al.*, 2010). There is only a single Group I WRKY gene in the green algae *Chlamydomonas reinhardtii*, the non-photosynthetic slime mold *Dictyostelium discoideum*, and the unicellular protist *Giardia lamblia*, suggesting that Group I WRKY proteins with two WRKY domains are the ancestors to the other groups of WRKY proteins (Zhang and Wang, 2005). The ancestral Group I WRKY gene was likely to have been generated from domain duplication of a proto-WRKY gene with a single WRKY domain. Subsequent loss of the N-terminal WRKY domain of Group I WRKY genes led to Goup II and Group III WRKY genes with a single WRKY domain, with Group III genes being evolutionarily the last. However, comparative studies of more recently available genome sequences from the filamentous terrestrial alga *Klebsormidium flaccidum* and the spike moss *Selaginella moellendorffii* have raised questions about whether all WRKY genes evolved from an ancestral Group I WRKY gene (Rinerson *et al.*, 2015). Furthermore, both the spike moss *S. moellendorffii* and the moss *Physcomitrella patens* contain Group III or Group III-like WRKY genes but lack specific subgroups of Group II WRKY genes, suggesting that Group III WRKY genes are probably not the youngest group of WRKY genes (Rinerson *et al.*, 2015).

A large number of studies have established that plant WRKY transcription factors play critical roles in the two interconnected branches of the plant innate immune system: pathogen-associated molecular patterns (PAMP-triggered immunity or PTI) and pathogen virulent effectors (effector-triggered immunity or ETI) (Jones and Dangl, 2006). It has been widely reported that many WRKY genes from a wide range of plant species are responsive to pathogens or pathogen elicitors. In Arabidopsis, more than 70% of the WRKY gene family members are responsive to pathogen infection and salicylic acid (SA) treatment (Dong *et al.*, 2003). Studies using knockout or knockdown mutants or overexpression lines of WRKY genes have shown that WRKY transcription factors can positively or negatively regulate plant PTI and ETI (Chen and Chen, 2000, 2002; Li *et al.*, 2004, 2006; Eulgem, 2006; Kim *et al.*, 2006, 2008; Xu *et al.*, 2006; Zheng *et al.*, 2006, 2007; Eulgem and Somssich, 2007; Lai *et al.*, 2008, 2011; Pandey and Somssich, 2009; Mao *et al.*, 2011). Well-characterized signaling pathways leading to the induction or activation of specific WRKY proteins and their subsequent activation of downstream target genes in plant defense responses have also been reported. WRKY proteins also regulate plant responses to a broad spectrum of abiotic stresses including heat, salt, osmotic, and drought stress (Jiang *et al.*, 2012; Ding *et al.*, 2013, 2014, 2015; Hu *et al.*, 2013; Xiao *et al.*, 2013; Sun and Yu, 2015). Other studies have shown that WRKY proteins are involved in hormone signaling (Shang *et al.*, 2010; Yu *et al.*, 2010; Chen *et al.*, 2013; Jiang *et al.*, 2014; Ding *et al.*, 2015), secondary metabolism (Wang *et al.*, 2010; Suttipanta *et al.*, 2011) and other important processes associated with plant stress responses. There are also a number of reports on the role of WRKY proteins in the regulation of plant growth and developmental processes, including trichome (Johnson *et al.*, 2002) and seed development (Luo *et al.*, 2005), germination (Jiang and Yu, 2009), and leaf senescence (Robatzek and Somssich, 2002; Miao *et al.*, 2004). Thus, even though WRKY proteins have important roles in diverse biological processes in plants, their predominant functions appear to be the regulation of plant responses to a broad spectrum of biotic and abiotic stresses.

Among the large number of reported studies on the roles of WRKY transcription factors, the vast majority of them have been carried out in the model plants Arabidopsis and, to a less extent, rice, while only a relatively few studies have reported on WRKY proteins from other plants including important crop species. Comparative analyses of sequenced plant genomes have revealed a great variation in the number of WRKY genes in different plant species, due to differences in the frequencies in both large-scale genome duplications and small-scale gene duplications (Rinerson *et al.*, 2015). Furthermore, although WRKY homologs from different species are highly similar in the conserved WRKY domain regions, they can display substantial sequence divergence in other regions, which could lead to differences in their functions. Therefore, efforts in analyzing WRKY genes from non-model plants could lead to discoveries of new or even novel functions of this important superfamily of transcription factors. In the present study, we report a functional analysis of two structurally related soybean WRKY proteins, GmWRKY58 and GmWRKY76, in plant growth and development. GmWRKY58 and GmWRKY76 are close homologs of Arabidopsis AtWRKY70 and AtWRKY54, two well-characterized Group III WRKY proteins with a critical role in plant defense and stress responses (Li *et al.*, 2004, 2006, 2013; Knoth *et al.*, 2007; Besseau *et al.*, 2012; Hu *et al.*, 2012; Shim and Choi, 2013; Shim *et al.*, 2013). However, unlike AtWRKY70 and AtWRKY54, overexpression of *GmWRKY58* or *GmWRKY76* in transgenic Arabidopsis plants had no major effect on disease resistance or abiotic stress tolerance but could substantially promote flowering and increased expression of flowering-related genes of transgenic plants. Most importantly, virus-induced silencing of *GmWRKY58* and *GmWRKY76* in soybean caused severe stunted growth with reduced size of leaves and plant stature. The critical role of the two soybean WRKY genes in plant growth and development is consistent with their patterns of elevated expression in relatively young leaves of soybean plants and their binding to the promoters of some flowering-related genes from Arabidopsis. These results strongly suggest that close structural homologs of WRKY proteins from different plant species can evolve into functionally divergent WRKY proteins that regulate distinct biological processes in plants.

## Materials and methods

### Plant material and growth conditions

Soybean (*Glycine max* cv ‘Williams 82’) and tobacco plants were grown in a greenhouse or a growth room at 25 °C with a 12/12h hour light/dark photoperiod. Arabidopsis plants were grown at 24 °C light/22 °C dark with a 12/12h photoperiod.

### Identification and phylogenetic analysis of Group III proteins

Phylogenetic analysis was performed using MEGAv5. The full sequences of Group III WRKYs in Arabidopsis, rice, and soybean (see Supplementary Table S1 at *JXB* online) were used to construct multiple sequence alignments using Clustal W. A phylogenetic tree was produced following neighbor-joining method using the aligned sequences.

### Production of recombinant proteins and electrophoretic mobility shifting assay (EMSA)

For generation of GmWRKY58 and GmWRKY76 recombinant protein, their full-length cDNAs were PCR-amplified using gene-specific primers (5′- AGCGGATCCATGAGTATTCTCTTCCC AAGAAGT-3′ and 5′- AGCAAGCTTCTAAAGCAATTGGCTTT CATCAAAG -3′), cloned into pET32a (Novagen, San Diego, CA, USA). The cloned *GmWRKY58* and *GmWRKY76* genes in the recombinant pET32a vector were confirmed by sequencing, and transformed into *E. coli* strain BL21 (DE3). Induction of protein expression and purification of recombinant His-tagged proteins were performed according to the protocol provided by Novagen. The recombinant proteins were purified according to the Novagen manual. Oligo nucleotides were labeled using the Biotin 3′ End DNA Labeling Kit (Thermo Scientific, Waltham, MA, USA) according to the manufacturer’s instructions. A DNA binding assay was performed based on the Light Shift Chemiluminescent EMSA Kit (Thermo Scientific, Waltham, MA, USA).

### Assays of transcription-activating activity in yeast and plant cells

The full-length coding sequences of *GmWRKY58* and *GmWRKY76* were PCR-amplified using gene-specific primers (5′-AGCGAATTCA TGAGTATTCTCTTCCCAAGAAGT-3′ and 5′-AGCGTCGA CGCTAAAGCAATTGGCTTTCATCAAAG-3′) and cloned into pBD-Gal4Cam to generate translational in-frame fusion with the DNA-binding domain of GAL4 transcription factor. The pBD-WRKY fusion constructs were transformed into yeast strain YRG-2. Transcription-activating activity of GmWRKY58 and GmWRKY76 in yeast cells were determined by the *LacZ* reporter gene expression using assays of β-galactosidase activity. The empty pBD-Gal4Cam vector was used as a control.

The transcriptional regulatory activities of GmWRKY58 and GmWRKY76 in plant cells were determined using a previously described GUS reporter gene system (Kim *et al.*, 2006). The GUS reporter gene is driven by a synthetic promoter consisting of the –100-nucleotide minimal CaMV 35S promoter and eight copies of the *LexA* operator sequence (Kim *et al.*, 2006). For generating effector genes, the DNA fragment for the DNA-binding domain (DBD) of LexA was fused with *GmWRKY58* and *GmWRKY76* coding sequences and the translational fusion genes were cloned into the pTA7002 vector behind the glucocorticoid-inducible promoter (Aoyama and Chua, 1997). As control, the unfused *LexA DBD*, *GmWRKY58*, and *GmWRKY76* genes were also cloned into pTA7002. Arabidopsis plant protoplasts were prepared from 4-week-old Arabidopsis leaves using the tape-Arabidopsis sandwich method (Wu *et al.*, 2009) and transfected with the effector constructs a combination with the GUS reporter construct. Transfected protoplasts with each effector/report combination were divided into two equal aliquots and dexamethasone (DEX) was added into one of the aliquots to a final concentration of 5 µM to induce expression of the effector gene. After 20h, protoplasts were harvested and the GUS activities from transfected protoplasts with or without DEX treatment were determined and compared. GUS activity was measured through a 4-methylumbellifery-β-D-glucuronide substrate assay.

### Subcellular localization

DNA fragments for full-length or N-terminal domains of GmWRKY58 and GmWRKY76 proteins were PCR-amplified using gene-specific primers (5′-AGCGAGCTCATGAGTATT CTCTTCCCAAGAAGT -3′ and 5’-AGCGGATCCAAGCAATT GGCTTTCATCAAAG -3′) and fused to a GFP plasmid. Correct sequences and fusion of the constructs were confirmed by DNA sequencing.

*Agrobacterium* cells containing the GFP fusion constructs were introduced into leaves of transgenic *Nicotiana benthamiana* that expresses a histone 2B fused with a red fluorescent protein (RFP-H2B) as a marker for the nucleus (Yang *et al.*, 2011). Infected leaves were analyzed at approximately 24h after infiltration. Images were observed by confocal laser microscopy.

### Analysis of gene expression using qRT-PCR

Soybean tissue samples were lyophilized and stored at –80 °C until use. Total RNA was isolated from soybean tissues using the Trizol reagent according to the supplier**’**s instruction. Extracted RNA was treated with DNase to remove contaminating DNA and reverse transcribed using the ReverTran Ace^®^ qPCR RT kit (Toyobo) for reverse transcriptase-PCR. qRT-PCR was performed with an StepOnePlus™ Real-Time PCR System (ABI). PCRs were performed using the SYBR^®^ Green qPCR Master Mixes (Takara) and gene-specific primers (see Supplementary Table S2). The PCR conditions consisted of denaturation at 95 °C for 1min, followed by 40 cycles of denaturation at 95 °C for 15s, annealing and extension at 58 °C for 30s. Melt curve analysis was performed on the end products of PCR, to determine the specificity of reactions. Relative quantification of gene expression was calculated according to the ΔΔCt method. The soybean *Actin* gene (Gm18g52780) was used as internal control.

### Generation of transgenic *GmWRKY58* and *GmWRKY76* Arabidopsis plants

For generating transgenic *GmWRKY58*- and *GmWRKY76*-expressing Arabidopsis plants, the full-length coding sequences were amplified using gene specific primers (5′-AGCGTCGACATG AGTATTCTCTTCCCAAGAAGTTC-3′ and 5′-AGCGGATCCCT AAAGCAATTGGCTTTCATCAA-3′). The amplified fragments were digested using appropriate restriction enzymes and inserted into the plant transformation vector pFGC5941 containing the CaMV *35S* promoter. The fused plasmids were transformed into Col-0 wild-type plants using the *Agrobacterium*-mediated floral-dip procedure (Clough and Bent, 1998). Transformants were identified for resistance to Basta herbicide. Transgenic plants overexpressing the transformed *GmWRKY58* and *GmWRKY76* transgene were identified using RT-qPCR.

### Phenotypic analysis of transgenic Arabidopsis plants

Pathogen inoculations were performed by infiltration of leaves of 6 to 10 plants for each treatment with *Pseudomonas syringae* pv. tomato DC3000 (PstDC3000) (OD_600_=0.0001 in 10mM MgCl_2_). Inoculated leaves were harvested 3 d post inoculation and homogenized in 10mM MgCl_2_. Diluted leaf extracts were plated on King’s B medium supplemented with rifampicin (100 µg ml^–1^) and kanamycin (25 µg ml^–1^) and incubated at 25 °C for 2 d before counting the colony-forming units.

For determining plant tolerance to osmotic stress, 4-week-old plants were watered with 15% polyethylene glycol (PEG) 6000 solution for 3 d. Control plants received water. For electrolyte leakage determination, plant material (0.5g) was washed with deionized water and placed in tubes with 20ml of deionized water. The electrical conductivity of this solution (L1) was measured after 1h of shaking at room temperature. Then, the samples were boiled for 20min and measured a second time for conductivity (L2). The electrolyte leakage was calculated as follows: EL (%) = (L1/L2)×100%. For determination of germination, seeds of WT and transgenic plants were surface-sterilized and sown on half-strength MS medium containing varying concentrations of NaCl, mannitol or abscisic acid (ABA). The plates were incubated for 48h at 4 °C and then transferred to an incubator at 25 °C (12h day/night photoperiod). The germination rates were scored for 4 d after transfer to 25 °C.

### Chromatin immunoprepitation (ChIP)

For ChIP assays, we first generated the *35S::GmWRKY58-4myc* and *35S::GmWRKY76-4myc* constructs and Arabidopsis protoplasts were transfected with the construct DNA. After 24h, protoplasts were harvested and ChIP assays were conducted using anti-myc antibody or mouse IgG (negative control), as described previously (Mao *et al.*, 2011). qPCRs were performed on the immunoprecipitated DNA and input DNA using primer pairs specific for the promoter regions of flowering time genes (see Supplementary Table S3). The ChIP results are shown as percentage of input DNA.

### BPMV-mediated gene silencing

DNA fragments of *GmWRKY58* and *GmWRKY76* genes were PCR-amplified using gene-specific primers and cloned into BPMV RNA2 silencing vector (Zhang *et al.*, 2010, 2013). Inoculation of soybean seedlings at the V1 stage with DNA-based BPMV constructs via biolistic particle bombardments was performed as described previously (Zhang *et al.*, 2010, 2013). At 3 weeks post-virus inoculation, the third trifoliate leaves were harvested to isolate RNA for detection of BPMV by RT-PCR and verification of gene silencing by qRT-PCR.

## Results

### Protein structures of GmWRKY58 and GmWRKY76

Our study of soybean GmWRKY58 and GmWRKY76 originated from our interest in identifying those members of the soybean WRKY superfamily that are capable of enhancing pathogen resistance and stress tolerance when overexpressed in transgenic Arabidopsis plants. From the sequenced soybean genome, we initially identified more than 170 WRKY genes and analyzed their expression in response to pathogens, abiotic stresses, and defense- and stress-related phytohormones such as salicylic acid (SA) and ABA. From the expression analysis, we identified more than 100 defense/stress-responsive soybean WRKY genes. To analyze more directly their ability to affect plant defense and stress responses, we generated overexpression constructs under the strong CaMV *35S* promoter for the stress/defense-responsive soybean WRKY genes and transformed some of them into the model plant *Arabidopsis thaliana*. Transgenic Arabidopsis plants overexpressing the vast majority of the stress-responsive soybean WRKY genes displayed no significant alteration in plant morphology, growth, and development. As will be described later in detail, however, transgenic plants overexpressing soybean *GmWRKY58* and *GmWRKY76* were not altered in plant disease resistance or stress tolerance but were substantially altered in flowering time when compared to control plants, suggesting their specific role in plant growth and development.

**Fig. 1.**
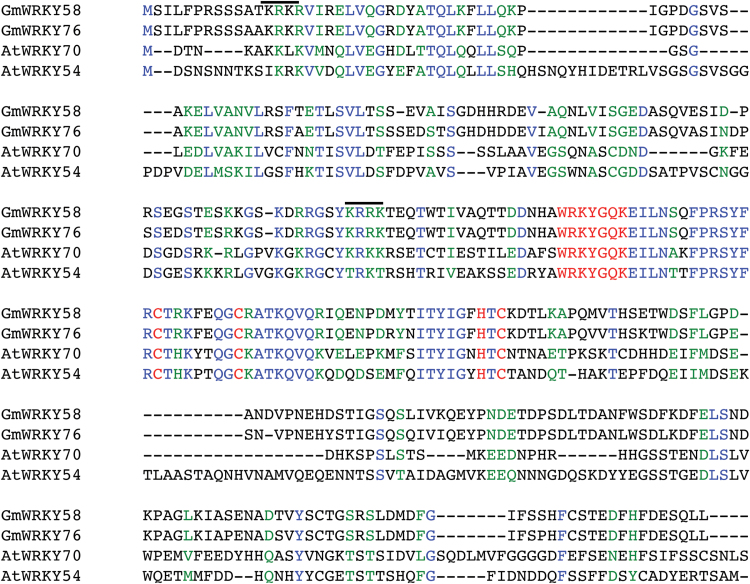
Sequence alignment of GmWRKY58 and GmWRKY76 with the AtWRKY70 and AtWRKY54 homologs from Arabidopsis. Amino acids identical in four proteins are blue, and residues similar in four proteins are green. The WRKY DNA binding motifs with the highly conserved WRKYGQK sequences and the residues forming the C2HC zinc-fingers are red. Putative nuclear localization signals are indicated by bars.

GmWRKY58 and GmWRKY76 are close homologs that share 91% identical amino acid residues (Fig. 1). Both GmWRKY58 and GmWRKY76 are Group III WRKY proteins, each containing a single WRKY domain with a C2HC zinc finger structure (Fig. 1). A BLAST search against the 72 identified Arabidopsis WRKY proteins showed that GmWRKY58 and GMWRKY76 are most closely related to AtWRKY70 and AtWRKY54 (Fig. 1), two of the most analyzed Arabidopsis WRKY proteins with critical roles in plant responses to both biotic and abiotic stresses (Li *et al.*, 2004, 2006, 2013; Knoth *et al.*, 2007; Besseau *et al.*, 2012; Hu *et al.*, 2012; Shim and Choi, 2013; Shim *et al.*, 2013). As expected, the two soybean WRKY paralogs shared the highest homology with the two Arabidopsis WRKY proteins in the DNA-binding WRKY domain, but substantial sequence similarities were also found in the N-terminal regions of the proteins (Fig. 1). On the other hand, the sequences of GmWRKY58 and GmWRKY76 are highly divergent from those of AtWRKY70 and AtWRKY54 in the C-terminal region. No other known structural motifs were identified from GmWRKY58 or GmWRKY76, apart from the highly conserved WRKY domain.

**Fig. 2.**
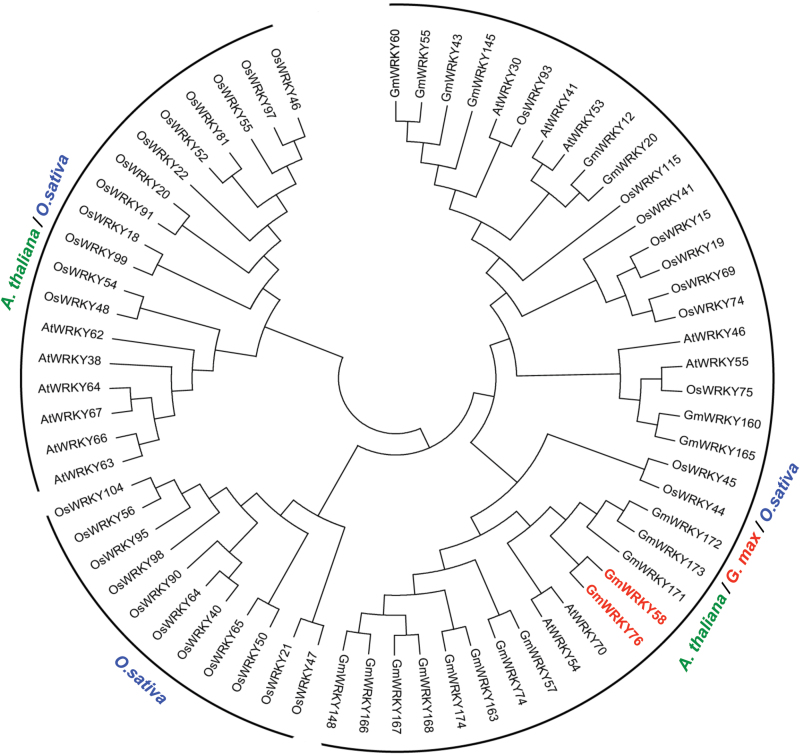
Phylogenetic analysis of Group III WRKY proteins from Arabidopsis, soybean, and rice. The full sequences of group III WRKYs in Arabidopsis, rice, and soybean were used to construct multiple sequence alignments using Clustal W. The phylogenetic tree was produced with the aligned sequences following the neighbor-joining method using MEGAv5. Three subclades with proteins from one, two, and all three plant species are indicated. GmWRKY58 and GmWRKY76 are highlighted on the tree in red.

To further analyze the structural relationship of GmWRKY58 and GmWRKY76 with other Group III WRKY proteins, we generated a maximum-likelihood phylogenetic tree of Group III WRKY proteins from Arabidopsis, soybean, and rice. The tree contains subclades with proteins from all three plant species or subclades with proteins exclusively from one or two plant species (Fig. 2). Most notably, a subclade consists of Group III WRKY proteins exclusively from rice, most likely reflecting the monocot nature of rice and thus evolutionarily more distantly related to the two dicot plants (Fig. 2). In addition, the phylogenetic analysis confirmed that soybean GmWRKY58 and GMWRKY76 are structurally closely related to Arabidopsis AtWRKY70 and AtWRKY54 (Fig. 2). Three other soybean Group III proteins, GmWRKY171, GmWRKY172, and GmWRKY173, are also structurally closely related to GmWRKY58 and GmWRKY76 (Fig. 2). These three Group III WRKY proteins are encoded by three clustered genes on Chromosome 14, most likely resulting from local gene duplication.

### DNA binding and transcription-activating activities and subcellular localization

**Fig. 3.**
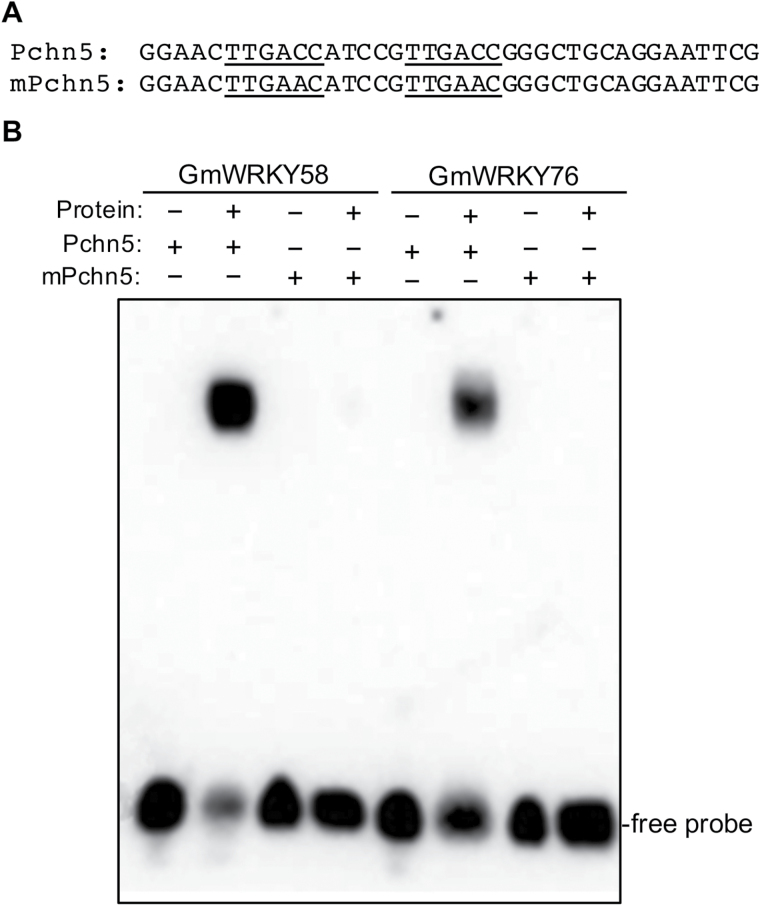
W-box-binding activities of GmWRKY58 and GmWRKY76. (A) Nucleotide sequences of probes used for DNA binding assays. Pchn5 contains two TTGACC W-box sequences, which are mutated into TTGAAC in mPchn5. (B) Recombinant GmWRKY58 and GmWRKY76 were purified from *E. coli* cells and used for DNA binding assays with Pchn5 and mPchn5 as probes. The binding reactions (20 µl) contained 2ng labeled oligo DNA, 5 µg polydeoxyinosinic-deoxycytidylic acid, and 1 µg recombinant protein. The binding assays were repeated twice with independently prepared recombinant proteins with similar results.

WRKY transcription factors recognize the TTGACC/T W-box sequences, which occur at high frequencies in the promoters of defense/stress-induced genes (Dong *et al.*, 2003). To examine the DNA-binding activity of GmWRKY58 and GmWRKY76, we expressed the genes in *E. coli*, purified the recombinant proteins, and analyzed their binding to an oligonucleotide that contains two direct TTGACC repeats (Pchn5; Fig. 3A) using an electrophoretic mobility shifting assay (EMSA). A protein/DNA complex with a reduced mobility was detected when purified recombinant GmWRKY58 or GmWRKY76 protein was incubated with the Pchn5 probe (Fig. 3B). Binding of GmWRKY58 or GmWRKY76 was not detected with a mutant probe (mPchn5) in which both TTGACC sequences were changed to TTGAAC (Fig. 3). Thus, binding of the two soybean Group III WRKY proteins to the TTGACC W-box sequence is highly specific.

**Fig. 4.**
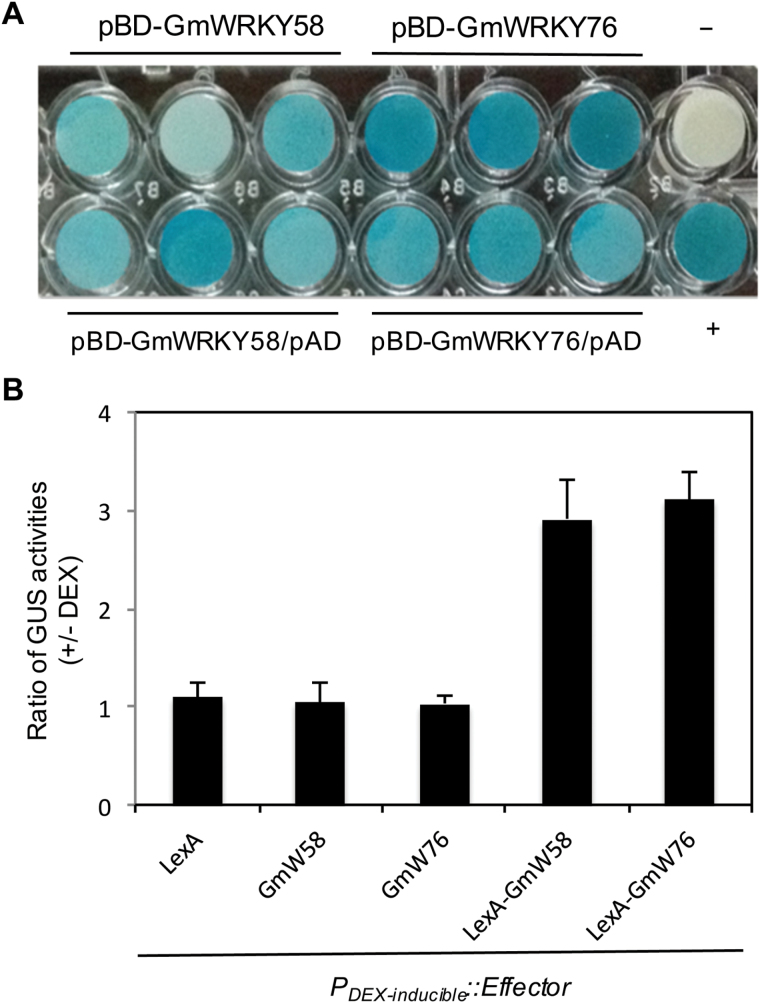
Transcription-activating activities of GmWRKY58 and GmWRKY76. (A) The pBD-GmWRKY58 and pBD-GmWRKY76 fusion vectors were transformed alone (upper row) or in combination with a pAD empty vector (lower row) into yeast cells. Activation of the LacZ reporter gene in the yeast cells was determined by assays of the β-galactosidase activity with X-gal as substrate. Empty pBD (–) and pBD-WT/pAD-WT (+) were used as negative and positive controls, respectively. (B) Arabidopsis leaf protoplasts were transfected with the construct of a GUS reporter gene under the control of a promoter containing copies of the *LexA* operator sequence in combination with an effector gene of LexA DBD (LexA), GmWRKY58, GmWRKY76, LexA DBD-GmWRKY58 (LexA-GmW58), or Lex ADBD-GmWRKY76 (LexA-GmW76) under the control of a DEX-inducible promoter in the pTA7002 vector. Transfected protoplasts were divided into two aliquots, to one of which was added 5 µM DEX to induce expression of the effector genes. GUS activities from transfected protoplasts with or without DEX treatment were determined 20h later and compared. GUS activity was measured through a 4-methylumbellifery-β-D-glucuronide substrate assay.

We also analyzed the transcriptional activating activity of GmWRKY58 and GmWRKY76 in both yeast and plant cells. The protein-coding regions of the *GmWRKY58* and *GmWRKY76* cDNAs were inserted downstream of the GAL4 DNA-binding domain (DBD) in a yeast transformation plasmid. These fusion constructs were then introduced into the yeast strain YRG-2, in which two reporter genes (*HIS*3 and *Lac*Z) are under the control of the GAL4 binding sites. As shown in Fig. 4A, yeast cells transformed with the fusion construct contained high levels of β-Gal activity. These yeast cells were also able to grow on media without histidine (data not shown). These results indicate that the fusion proteins of GmWRKY58 and GmWRKY76 are capable of activating both *HIS*3 and *Lac*Z reporter genes and, therefore, are transcriptional activators in yeast cells. The transcriptional regulatory activities of GmWRKY58 and GmWRKY76 in plant cells were determined using a previously described GUS reporter gene system (Kim *et al.*, 2006). The GUS reporter gene is driven by a synthetic promoter consisting of the –100-nucleotide minimal CaMV *35S* promoter and eight copies of the *LexA* operator sequence. To generate effector genes, we fused the *LexA DBD* sequence with those of *GmWRKY58* and *GmWRKY76* coding sequences in pTA7002 vector behind its steroid-inducible promoter (Aoyama and Chua, 1997). Unfused LexADBD, GmWRKY58, and GmWRKY76 genes were also cloned into pTA7002 as negative controls. Arabidopsis leaf protoplasts were transfected with different effector/reporter combinations and changes in GUS reporter activity as a result of DEX-induced expression of introduced effector genes were determined. As shown in Fig. 4B, induced expression of unfused *LexA DBD*, *GmWRKY58*, and *GmWRKY76* genes did not affect the co-transfected GUS reporter gene expression. By contrast, induced expression of fused *LexA DBD-GmWRKY58* and *LexA DBD-GmWRKY76* increased the GUS reporter activities by 2-fold. These results indicate that both GmWRKY58 and GmWRKY76 function as transcriptional activators in plant cells.

**Fig. 5.**
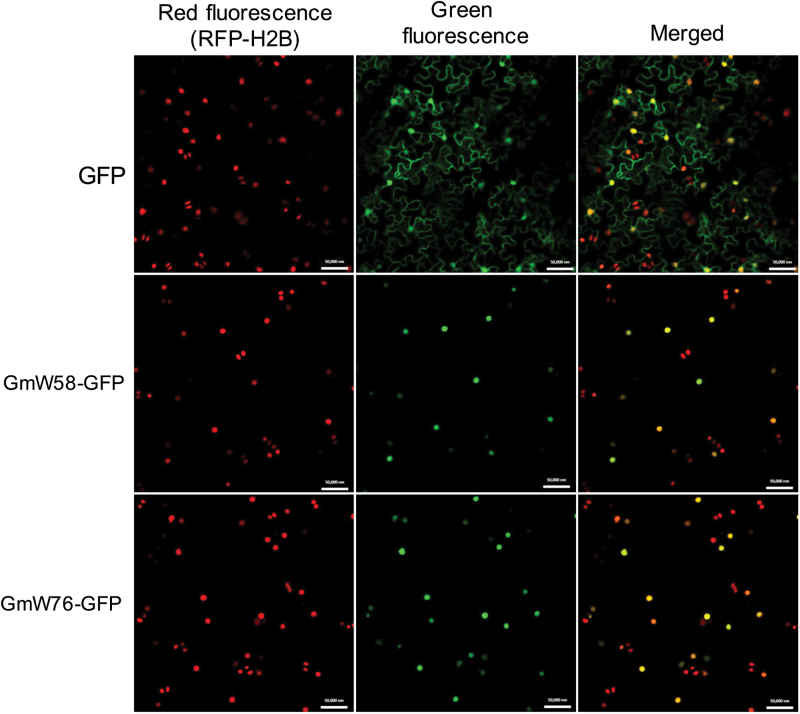
Subcellular localization of GmWRKY58 and GmWRKY76. The GmWRKY58- and GmWRKY76-GFP fusion genes were expressed transgenic *N. benthamiana* plants expressing a H2B-RFP nuclear marker. A majority of GmWRKY58- and GmWRKY76-GFP punctate fluorescence signals were also labeled by the H2B-RFP nuclear marker signals. Expression of unfused GFP generated both dispersed and punctate signals. Bars=10 µm.

If GmWRKY58 and GmWRKY76 function as transcription factors, they are likely to be localized in the nucleus. At least two putative nuclear localization signals are present in GmWRKY58 and GmWRKY76 based on prediction by the PSORT II program (Fig. 1). To determine their subcellular location, we constructed GFP protein fusion of the two soybean WRKY proteins. The fusion constructs, driven by the CaMV *35S* promoter, were agroinfiltrated for transient expression into transgenic tobacco (*Nicotiana benthamiana*) containing the nuclear marker histone 2B fused with red fluorescent protein (RFP-H2B) and confocal microscopy was performed on the leaf sections of agroinfiltrated plants. As shown in Fig. 5, the transiently expressed GmWRKY58-GFP and GmWRKY76-GFP fusion proteins were localized exclusively in the nucleus of tobacco cells. By contrast, GFP was found in both the nucleus and cytoplasm due to its small size.

### Expression of GmWRKY58 and GmWRKY76

**Fig. 6.**
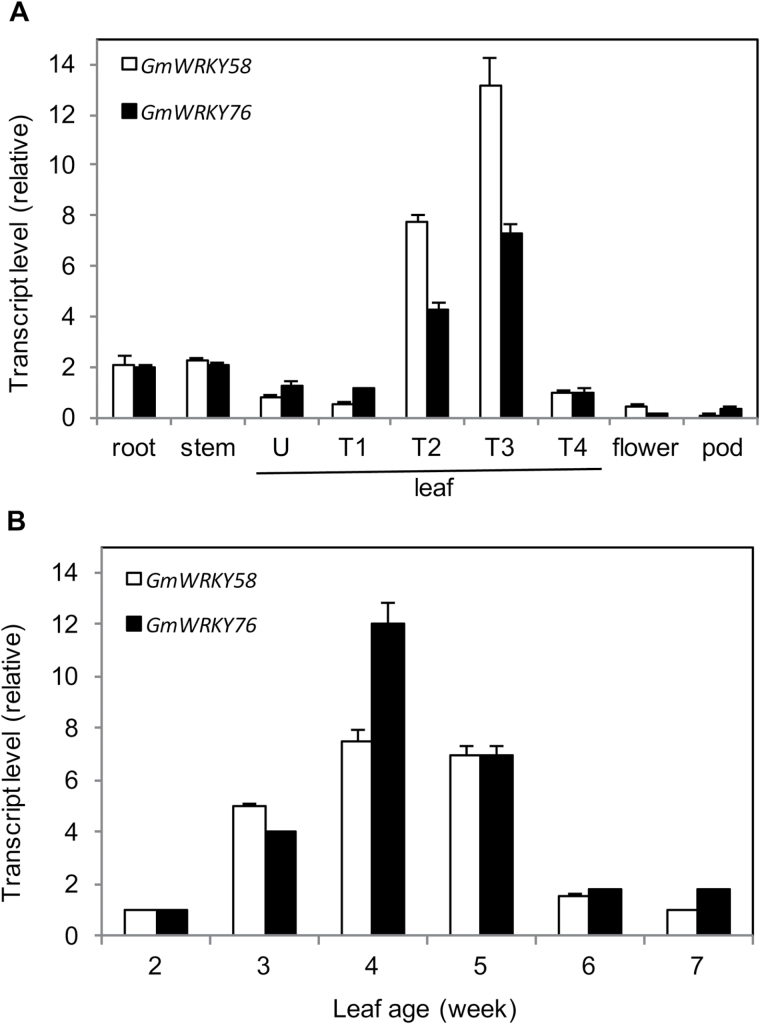
Tissue- and age-associated expression of *GmWRKY58* and *GmWRKY76*. (A) Expression of *GmWRKY58* and *GmWRKY76* in different plant tissues. Roots, stems, and leaves (U, unifoliate; T1, 1st trifoliate; T2, 2nd trifoliate; T3, 3rd trifoliate; T4, 4th trifoliate) were collected from seedlings when the third trifoliate leaves were fully expanded. Flowers were sampled from plants at reproductive 2 stage when the flowers were blooming, and pods were collected at 20-d post-flowering. (B) Age-regulated expression of *GmWRKY58* and *GmWRKY76* in soybean leaves. The first trifoliolate leaves were collected from 2-week-old seedlings to 7-week-old plants at 1-week intervals. *GmWRKY58* and *GmWRKY76* expression were analyzed by quantitative qRT-PCR using a soybean actin gene as an internal control. Values represent the means and standard errors of three replicates.

To further characterize GmWRKY58 and GmWRKY76, we examined their expression in soybean using qRT-PCR. First, we examined the transcript levels of the two WRKY genes in different soybean tissues. As shown in Fig. 6A, expression of both WRKY genes was detected in all tissues examined but the expression levels in roots, stem, and leaves were substantially higher than those in flowers and pods. We also compared unifoliate and trifoliate leaves for expression of the two WRKY genes and, interestingly, while transcripts for the two genes were detected in all these leaves, they were substantially higher in the second and third trifoliate leaves (Fig. 6A). This result suggests that expression of the genes in soybean leaves is developmentally regulated. To confirm this, we also examined the changes of transcripts levels of the two WRKY genes in the first trifoliate leaves of different ages. The expression of the genes was very low at young ages (2 weeks after germination), but steadily increased with age (Fig. 6B). By the 4th week after germination, the expression levels were about 8–12 times higher than those detected 2 weeks earlier (Fig. 6B). After peaking around the 4th week, the transcript levels of *GmWRKY58* and *GmWRKY76* declined steadily with further increased age, returning close to the basal levels by the 6th week. Thus, expression of both *GmWRKY58* and *GmWRKY76* in leaves started at very low levels, increased steadily in expanding leaves, remained highly expressed in relatively young expanded leaves, but then declined in old expanded leaves.

**Fig. 7.**
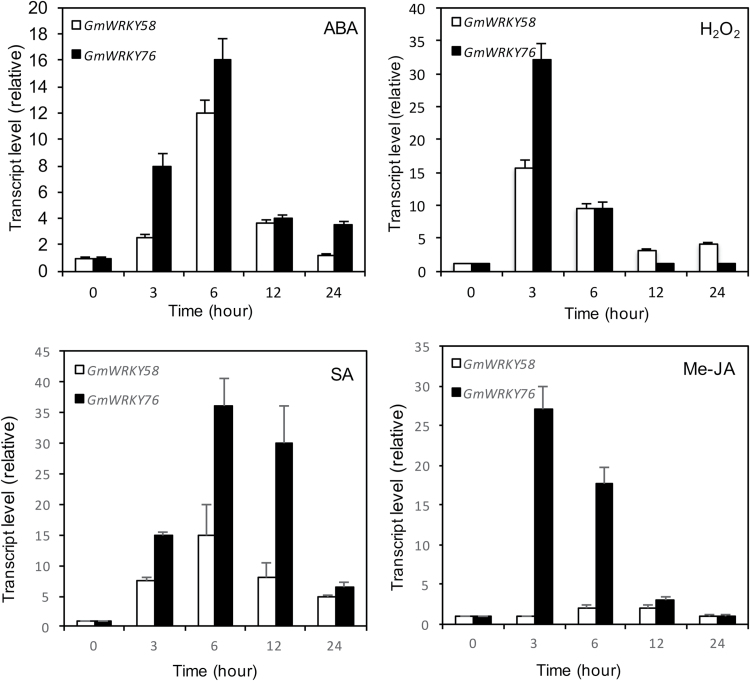
Expression of *GmWRKY58* and *GmWRKY76* in response to ABA, H_2_O_2_, salicylic acid (SA), and methyl JA (MeJA). The first trifoliolate leaves of 2.5-week-old soybean seedlings were treated with 100 μM ABA, 5mM H_2_O_2_, 2mM SA, or 100 µM MeJA. Leaf samples were collected at indicated time points for total RNA isolation, and gene expression was analyzed by qRT-PCR. Values represent the means and standard errors of three replicates.

Arabidopsis AtWRKY70 and AtWRKY54 are responsive to a range of biotic and abiotic stimuli (Li *et al.*, 2013). To determine the possible involvement of GmWRKY58 and GmWRKY76 in plant stress responses, we analyzed their expression in response to ABA, H_2_O_2_, SA, and methyl jasmonic acid (Me-JA). As shown in Fig. 7, both soybean WRKY genes were induced by ABA. By the 6th hour after ABA treatment, the transcript levels of the two WRKY genes were induced by more than 10-fold. By the 12+^th^ hour after ABA treatment, however, the transcript levels of the two WRKY genes had already declined substantially from the peak levels. Both *GmWRKY58* and *GmWRKY76* transcript levels were also elevated greatly upon H_2_O_2_ treatment and the kinetics of the induction, both in magnitude and speed, appeared to be stronger than with ABA treatment (Fig. 7). However, this induction of *GmWRKY58* and *GmWRKY76* by H_2_O_2_ was also transient, as evident from their rapid decline from the peak levels at the 3rd hour after treatment. Expression of *GmWRKY58* and, to a less extent, GmWRKY76 was also elevated in SA-treated plants (Fig. 7). Treatment with JA also greatly induced expression of GmWRKY76 but only marginally elevated the expression of GmWRKY58 (Fig. 7). Thus, *GmWRKY58* and *GmWRKY76* were responsive to the stress hormones and to H_2_O_2_.

### Promotion of flowering by GmWRKY58 and GmWRKY76 in Arabidopsis

**Fig. 8.**
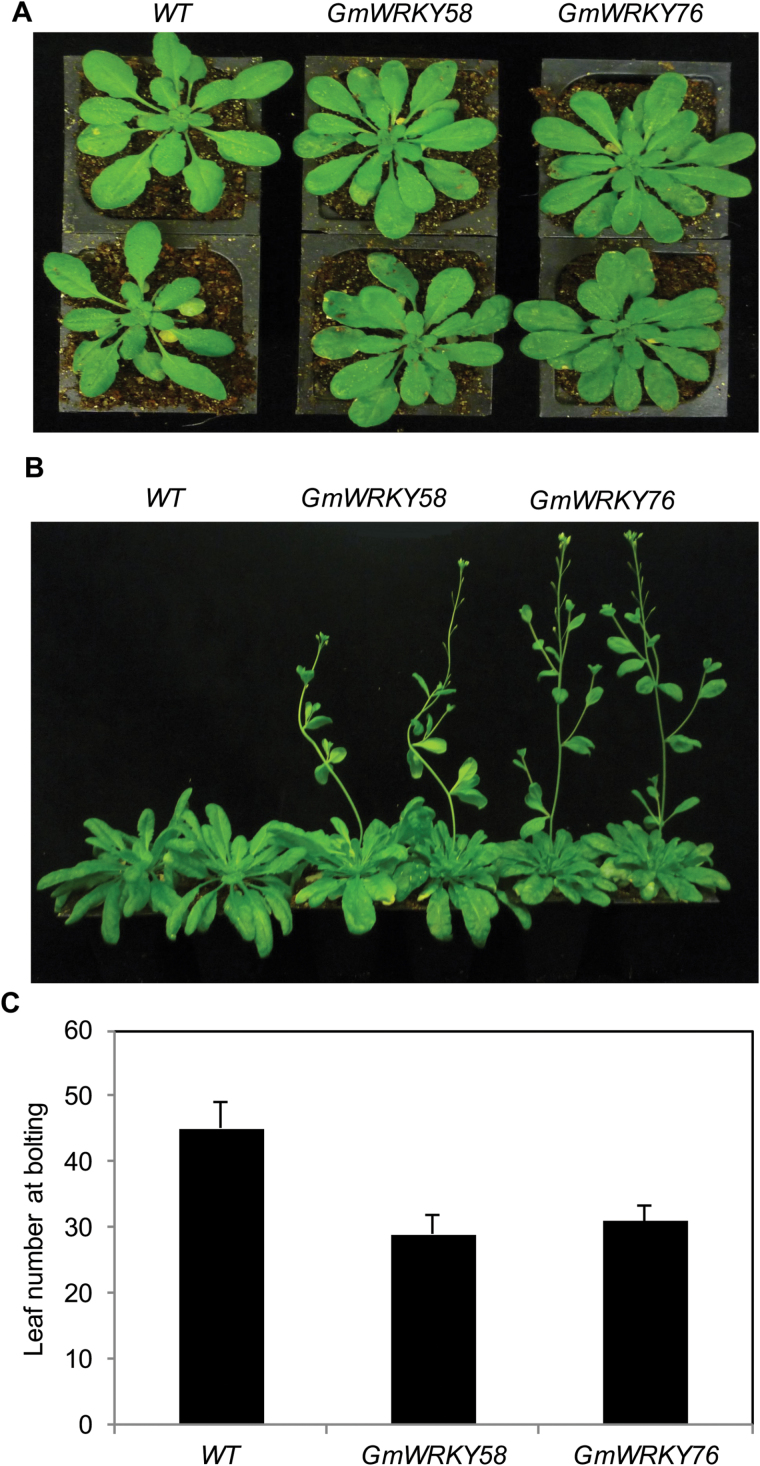
Overexpression of *GmWRKY58* and *GmWRKY76* and promotion of flowering in Arabidopsis. Transgenic Arabidopsis plants overexpressing *GmWRKY58* and *GmWRKY76* were identified by qRT-PCR (Supplementary Fig. 1). Homozygous T3 progeny of transgenic GmWRKY58 (lines 3 and 4) and GmWRKY76 (lines 1 and 3) were grown at 12/12h light/dark photoperiod. Images of representative plants were taken at 6 (A) and 8 (B) weeks after germination. The rosette leaf numbers at bolting were counted from 30 plants of two independent lines for each transgene and are shown as means and standard errors (C).

As described earlier, we were interested in functionally analyzing a large number of stress-responsive soybean WRKY genes through overexpression in Arabidopsis. Functional analysis of genes from crop plants, including soybean, through overexpression in Arabidopsis is a common and widely reported approach as the technique for transformation of the model plant is efficient and easy. In addition, many Arabidopsis WRKY genes have been well characterized and their close sequence similarity to many WRKY genes from soybean allows for comparative functional analysis. To analyze soybean WRKY genes in Arabidopsis, we first generated overexpression constructs of stress-responsive soybean WRKY genes under control of the constitutive and strong CaMV *35S* promoter. Overexpression constructs for more than 60 stress-responsive soybean WRKY genes were then transformed into Arabidopsis using the floral-dip procedure and transformants were identified for their herbicide resistance. From independent transgenic Arabidopsis plants for each of >60 soybean WRKY genes examined at the same time, only those transgenic plants transformed with the *GmWRKY58* and *GmWRKY76* overexpression constructs flowered substantially earlier than the control plants. Because of the importance of this phenotype, these transgenic *GmWRKY58* and *GmWRKY76* plants were further characterized for possible alterations in growth, development, and stress responses. From the >10 independent transgenic plants for either of the two soybean WRKY genes, a majority of them had the early flowering phenotype and expressed high levels of the soybean WRKY transgenes based on qRT-PCR analysis (see Supplementary Fig. S1). Homozygous progeny containing a single transgene locus from these *GmWRKY58*- or *GmWRKY76*-expressing lines were subsequently obtained and used for phenotypic characterization. As shown in Fig. 8A, these transgenic *GmWRKY58*- or *GmWRKY76*-expressing plants grew mostly normally without major morphological abnormalities in the vegetative stages. However, these plants flowered substantially earlier than control plants (Fig. 8B). The numbers of leaves at the time of bolting in these transgenic plants were only about 60% of those in wild-type plants (Fig. 8C).

**Fig. 9.**
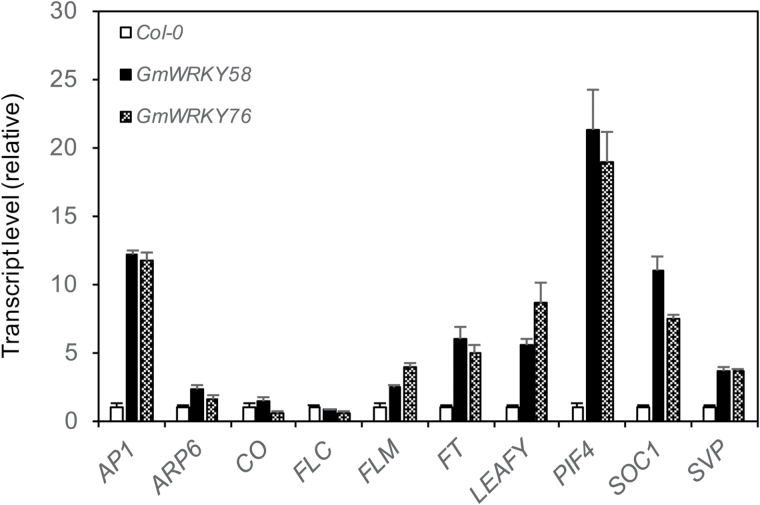
Flowering-related gene expression in Arabidopsis plants overexpressing *GmWRKY58* and *GmWRKY76*. The leaf samples were collected from 3-week-old seedlings of control Col-0 and transgenic *GmWRKY58* and *GmWRKY76* plants for total RNA isolation. Expression of 10 flowering-related genes was analyzed by qRT-PCR using an Arabidopsis actin gene as an internal control. Values represent the means and standard errors of three replicates.

To determine the molecular basis for the early flowering phenotype of the transgenic GmWRKY58 and GmWRKY76 Arabidopsis plants, we compared them with control plants for expression of a range of flowering-time genes in 3-week-old seedlings. These flowering-time genes include both positive and negative regulators (e.g. FLC) of plant flowering. As shown in Fig. 9, we observed increased expression for many of the flowering-promoting genes in the transgenic plants when compared to that in control plants. These genes include *AP1*, *FT*, *Leafy*, *PIF4*, *SOC1*, and *SVP1*. On the other hand, transcript levels of the flowering-inhibiting gene *FLC* were not significantly altered, while those for *FLM* were only increased slightly in the transgenic plants (Fig. 9). Thus, increased expression of these flowering-promoting genes even in the very young seedlings is consistent with the early flowering phenotypes of the transgenic *GmWRKY58* and *GmWRKY76* overexpression plants.

**Fig. 10.**
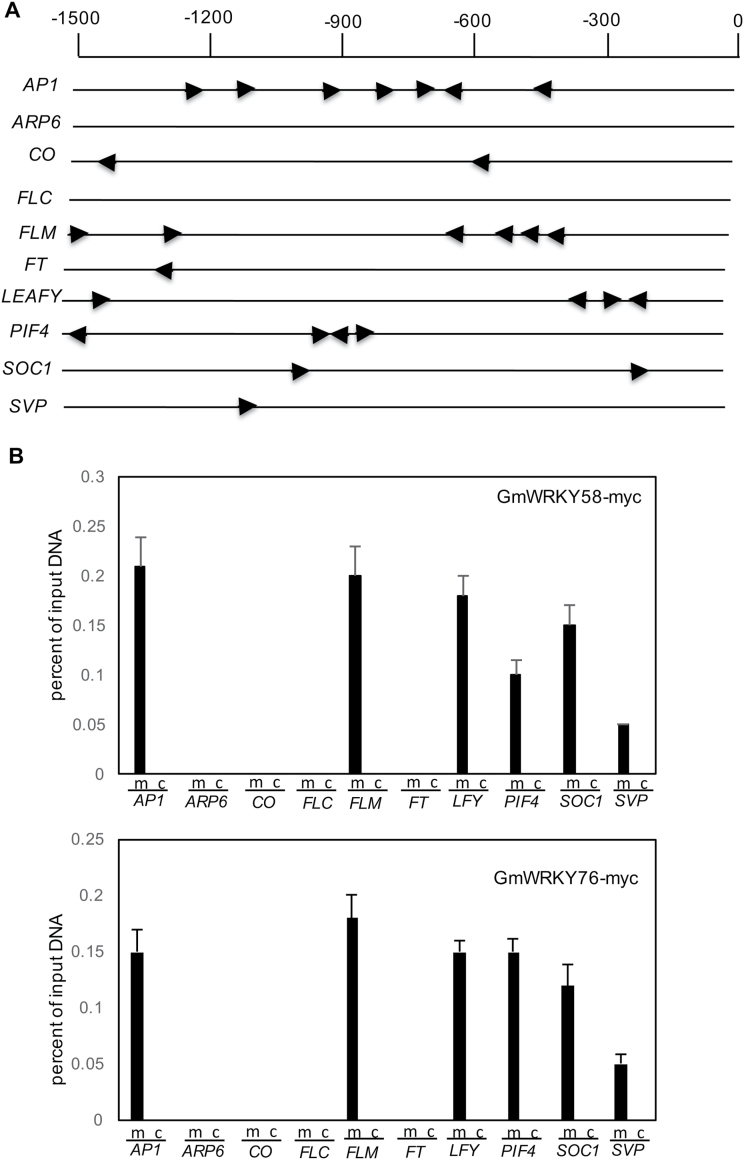
Binding of GmWRKY58 and GmWRKY76 to promoters of flowering-time genes. (A) W-box elements in the promoters of Arabidopsis flowering-related genes. Each W-box sequence (TTGAC) in the 1.5-kb promoter regions of the flowering-related genes is indicated with an arrow. Numbering is from the predicted translation start codons. (B, C) Arabidopsis leaf protoplasts were transfected with the *35S::GmWRKY58-4myc* or *35S::GmWRKY76-4myc* constructs. myc-Tagged GmWRKY-chromatin complexes were immunoprecipitated with an anti-myc antibody (m). A control reaction was processed side-by-side using mouse IgG (c). ChIP and input-DNA samples were quantified by real-time qPCR using primers specific to the promoters of the indicated flowering-time genes. The ChIP results are shown as a percentage of input DNA. Values represent the means and standard errors of three replicates.

As DNA-binding transcription factors, GmWRKY58 and GmWRKY76 promote flowering in transgenic plants most likely through regulation of transcription of genes that influence flowering either directly or indirectly. To determine the possibility of direct regulation of flowering-time genes by the two WRKY proteins, we examined the 1.5-kb region upstream of the translation start codon of these genes for the presence of the W-box sequences recognized by WRKY proteins. As shown in Fig. 10A, among the 10 flowering-time genes, only *ARP6* and *FLC* contain no TTGAC W-box core sequence. For the remaining flowering-time genes, *CO*, *FT*, *SOC1*, and *SVP* contain only 1 to 2 W-box elements. The other four genes (*AP1*, *FLM*, *LEAFY*, and *PIF4*) contain 4 to 7 W-box elements in their 1.5-kb promoter region. Interestingly, there appeared to be a generally positive, although not perfect, correlation between the number of W-box elements and the extent of their induction in the transgenic plants. For example, *ARP6* and *FLC* contain no W-box sequences and their expression was largely unaltered in the transgenic plants overexpressing *GmWRKY58* and *GmWRKY76*. On the other hand, *PIF4*, *AP1*, and *Leafy* are three of the four flowering-time genes containing the highest numbers of W-box sequences in their promoters (Fig. 10A) and they also displayed the highest induction in transgenic plants overexpressing *GmWRKY58* and *GmWRKY76* (Fig. 9). These results suggest that GmWRKY58 and GmWRKY76 may directly regulate the expression of at least some of the flowering-time genes. To test this possibility, we performed a ChIP-qPCR assay to determine whether GmWRKY58 and GmWRKY76 bind to the promoters of some of these flowering-time genes. As shown in Fig. 10B, C, promoters of *AP1*, *FLM*, *LFY*, *PIF4*, *SOC1*, and *SVP* were substantially enriched with an anti-myc antibody that immunoprecipitates myc-tagged GmWRKY58 and GmWRKY76 transgene products in transfected Arabidopsis protoplasts. By contrast, the IgG control failed to immunoprecipitate the promoter DNA of the six flowering-time genes. On the other hand, no significant enrichment of the promoters of *ARP6*, *CO*, *FLC*, or *FT* was detected after immunoprecipitation with the anti-myc antibody or the IgG control.

### Effects on disease resistance and stress tolerance by GmWRKY58 and GmWRKY76 in Arabidopsis

Arabidopsis AtWRKY70 has been extensively analyzed in plant defense responses. Analysis using both loss-of-function mutants and overexpression lines revealed that AtWRKY70 promotes SA-dependent defense pathways but suppresses JA-dependent signaling (Li *et al.*, 2004, 2006; Hu *et al.*, 2012; Shim and Choi, 2013; Shim *et al.*, 2013). To determine whether overexpression of GmWRKY58 and GmWRKY76 altered plant defense in transgenic plants, we analyzed the responses of Arabidopsis plants overexpressing the soybean WRKY genes to the virulent bacterial pathogen PstDC3000. A large number of studies have shown that Arabidopsis resistance to the virulent bacterial pathogen is dependent on SA signaling (Dong, 1998). When inoculated with the virulent bacteria, however, these transgenic *GmWRKY58* and *GmWRKY76* plants developed disease symptoms and supported bacterial growth at levels similar to those in control wild-type plants (see Supplementary Fig. S2). By contrast, the SA-signaling mutant *npr1* developed more severe disease symptoms and supported higher levels of bacterial growth when inoculated with the same virulent bacteria (Supplementary Fig. S2). Thus, overexpression of *GmWRKY58* and *GmWRKY76* did not affect SA-dependent defense against the bacterial pathogen in Arabidopsis plants.

Arabidopsis AtWRKY70 and its close homolog AtWRKY54 also modulate plant osmotic stress (Li *et al.*, 2013): both genes are induced by osmotic stress. Based on the assays using watering with 15% polyethylene glycol (PEG), it has been further found that the *atwrky70* and *atwrky54* mutant plants were more tolerant to osmotic stress (Li *et al.*, 2013). Likewise, expression of both *GmWRKY58* and *GmWRKY76* was responsive to ABA (Fig. 7), a phytohormone with an important role in plant stress responses. To determine further the roles of GmWRKY58 and GmWRKY76 in plants suffering from osmotic stress, we analyzed whether responses were affected by their overexpression. As previously described, we subjected the transgenic plants to osmotic stress by watering with 15% PEG6000 for 3 d, but observed no significant difference in the development of wilting symptoms between the overexpression lines and control wild-type plants (data not shown). There was no significant difference either in electrolyte leakage assayed on leaves after exposure to 15% PEG for 1 and 3 d (data not shown). We also compared their germination rates with those of control wild-type plants in response to salt (150mM NaCl), osmotic stress (300mM mannitol), and ABA (1 µM). As shown in Supplementary Fig. S3, no significant effects of overexpression of the two soybean WRKY genes on germination were observed in the presence of 300mM mannitol. On the other hand, during the first 2 d after sowing, seeds of the *GmWRKY58*- and *GmWRKY76*-overexpressing plants germinated at significantly higher rates than control plants in the presence of 150mM NaCl, but at lower rates in the presence of 1 µM ABA (see Supplementary Fig. S3). These differences, however, largely disappeared during the 3rd and 4th days. Thus, the effects of GmWRKY58 and GmWRKY76 on disease resistance and abiotic stress tolerance were relatively small.

### Stunted growth of soybean plants caused by silencing of *GmWRKY58* and *GmWRKY76*

**Fig. 11.**
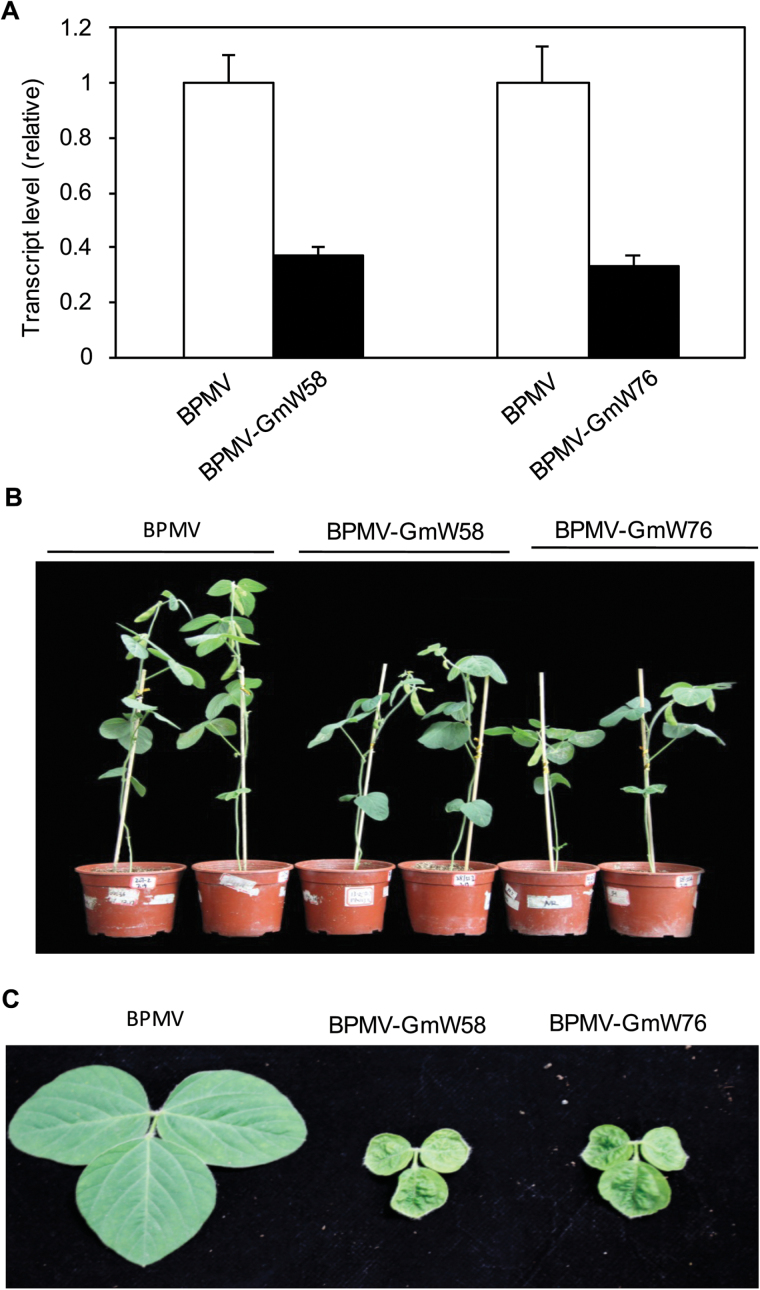
Silencing of *GmWRKY58* and *GmWRKY76* caused stunted growth. Expression of *GmWRKY Y58* and *GmWRKY76* was performed by qRT-PCR using a soybean actin gene as control (A). The results shown are from five individual *GmWRKY58*-silenced (BPMV-GmWRKY58) and *GmWRKY76*-silenced plants (BPMV-GmWRKY76) and empty BPMV vector control plants (BPMV-0). The pictures of representative plants (B) and trifoliate leaves (C) inoculated with empty vector (BPMV) or silencing BPMV-GmWRKY58 and BPMV-GmWRKY76 vectors were taken 7 weeks after inoculation. Some leaves were collected for RNA isolation, leaving only petioles in some plants shown in (B).

Unlike Arabidopsis, many plants including important crop species such as soybean are still relatively difficult to routinely and reliably transform. Virus-induced gene silencing (VIGS) provides an alternative approach for targeted down-regulation and functional analysis of genes. VIGS mediated by Bean pod mottle virus (BPMV) has been successfully used in the functional investigation of soybean genes involved in defense and other processes, including soybean MAPK4 (GmMPK4) (Liu *et al.*, 2011). To determine the role of GmWRKY58 and GmWRKY76 in soybean, we attempted to silence their gene expression using BPMV-mediated gene silencing. Gene fragments from *GmWRKY58* and *GmWRKY76* were separately cloned into the BPMV silencing vector and the viral vectors were delivered into soybean plants through particle bombardment. To determine the extent of silencing, we performed qRT-PCR of the transcript levels of the two soybean WRKY genes in inoculated plants. As shown in Fig. 11A, when compared to the plants inoculated with empty vectors, those inoculated with either *GmWRKY58*- or *GmWRKY76*-silencing vectors displayed a reduction of approximately 60% in the transcript levels of the targeted gene but also of the close homolog as well. As described earlier, *GmWRKY58* and *GmWRKY76* share a high nucleotide sequence identify (>90%) and as a result either the construct for silencing *GmWRKY58* or for silencing *GmWRKY76* can silence both genes. Plants in which *GmWRKY58* and *GmWRKY76* were silenced had consistent phenotypes characterized by stunted growth (Fig. 11B). The soybean cultivar used in the experiments (‘Williams 82’) is a Group III indeterminate variety. Under the growth conditions with 12/12h photoperiod, the control plants maintained vegetative growth even after flowering and pod setting. Those plants inoculated with *GmWRKY58*- and *GmWRKY76*-silencing vectors were normal during the first 3–4 weeks after inoculation. However, around 4 weeks after inoculation, those plants with severe silencing of *GmWRKY58* and *GmWRKY76* started to display stunted phenotypes characterized by slower emergence and smaller size of new leaves and shorter length of upper parts of the elongating stems (Fig. 11B, 11C). As a result, the stature of the upper part of plants with *GmWRKY58* and *GmWRKY76* silenced was substantially reduced, almost mimicking a determinate growth pattern, even though the stature of their lower parts was largely normal, with normal-sized leaves and pods.

## Discussion

As a large superfamily of plant transcription factors, WRKY proteins have been subjected to extensive functional analysis over the last two decades. The overwhelming majority of these studies have focused on the roles of WRKY transcription factors in broad plant responses to biotic and abiotic stresses. By contrast, a broad role of WRKY proteins in plant growth and development has not emerged, although a few WRKY proteins have been shown to play roles in certain specific plant developmental processes (Johnson *et al.*, 2002; Luo *et al.*, 2005, 2013; Li *et al.*, 2015). In the present study, we have provided strong evidence that two closely related soybean WRKY proteins, GmWRKY58 and GmWRKY76, have a critical role in plant growth and development. Even though they are close homologs of Arabidopsis AtWRKY70 and AtWRKY54, overexpression of GmWRKY58 and GmWRKY76 did not result in altered resistance to the bacterial pathogen *P. syringae* (see Supplementary Fig. S2) or in tolerance to osmotic stress – two phenotypes previously established for AtWRKY70 and AtWRKY54 (Li *et al.*, 2004, 2006; Hu *et al.*, 2012; Shim and Choi, 2013; Shim *et al.*, 2013). The effects of their overexpression on tolerance to salt and osmotic stress and sensitivity to ABA were also negligible, or relatively small and transient (Supplementary Fig. S3). On the other hand, transgenic plants overexpressing *GmWRKY58* and *GmWRKY76* flowered substantially earlier than control plants (Fig. 8), a phenotype not observed in AtWRKY70- and AtWRKY54-overexpressing plants. Furthermore, silencing of *GmWRKY58* and *GmWRKY76* led to stunted plants characterized by greatly inhibited growth of expanding leaves and elongating stems (Fig. 11). These results establish that these two closely related WRKY proteins play an important and specific role in plant growth and development.

In Arabidopsis, overexpressing of GmWRKY58 and GmWRKY76 had strong effects on flowering time but had no significance on vegetative growth such as leaf expansion (Fig. 8). On the other hand, silencing of the same soybean WRKY genes in soybean mostly affected the growth of vegetative organs (leaves and stems) (Fig. 11). This discrepancy of phenotypes could be due to the fact that the phenotypes in Arabidopsis resulted from the gain-of-function overexpression approach whilst those in soybean were from a loss-of-function approach through down-regulation of the targeted genes. The phenotypes of transgenic plants overexpressing a gene of interest are mostly likely indicative of the potential functions of the gene when it is expressed at high levels. On the other hand, the phenotypes of loss-of-function mutants or gene-silencing plants are more indicative of the biological functions of the gene when expressed at physiological levels. The discrepancy of phenotypes could also be due to differences between Arabidopsis and soybean in important aspects of growth and development. For example, when Arabidopsis plants switch from a vegetative to reproductive state, it leads to the formation of an inflorescence with a cluster of flowers arranged on the stem. On the other hand, when soybean cultivars such as ‘Williams 82’ used in our VIGS experiments start flowering, the shoot apical meristem remains vegetative, where it further regulates the production of lateral vegetative and reproductive growth (Tian *et al.*, 2010; Ping *et al.*, 2014). It is possible that GmWRKY58 and GmWRKY76 are capable of promoting the activity of terminal meristems, which in Arabidopsis could lead to earlier transition from vegetative growth to flowering. On the other hand, since terminal meristems of soybean remain vegetative even after flowers have already developed from the buds at the base, silencing of genes important for the activity of terminal meristems may primarily lead to compromised vegetative growth, causing stunted phenotypes as observed in *GmWRKY58*- and *GmWRKY76*-silenced soybean plants (Fig. 11). It should also be pointed that plant growth and development are functionally and mechanistically closely linked. Many factors including a variety of environmental cues and hormones can affect both growth and development. GmWRKY58 and GmWRKY76 could regulate certain genes with roles in both growth and development, but there might be differential effects of these genes in different plants due to differences in the regulation pathways and/or in the physiological states that affect growth and development.

As transcription factors, the critical roles of WRKY proteins in plant responses to biotic and abiotic stresses are mediated through their regulation of the transcription of a large number of genes involved in stress responses. Indeed, genes differentially regulated during the activation of plant defense responses such as SA-mediated systemic-acquired resistance are substantially enriched in the W-box sequences in their promoters (Maleck *et al.*, 2000). Both GmWRKY58 and GmWRKY76 bind W-box sequences (Fig. 3), act as transcriptional activators in both yeast and plant cells (Fig. 4), and are localized in the nucleus (Fig. 5). These properties make it highly likely that they positively regulate plant growth and development through transcriptional regulation of gene expression. Since both these WRKY proteins promote flowering in transgenic Arabidopsis, some of their potential target genes could be those controlling flowering. Indeed, earlier flowering of the transgenic *GmWRKY58*- and *GmWRKY76*-overexpressing plants was associated with increased expression of positive regulators of flowering including *AP1*, *FT*, *Leafy*, *PIF4*, *SOC1*, and *SVP1* (Fig. 9). A survey revealed the presence of W-box sequences in the promoters of all these flowering-promoting genes (Fig. 10A). The presence of a large number of W-box sequences in the promoters of some of these important flowering-controlling genes, including *AP1*, *LEAFY*, *FLM*, and *PIF4*, was also associated with their increased expression in the transgenic *GmWRKY58* and *GmWRKY76* plants (Figs 9 and 10A). In contrast, *FLC* and *ARP6* contain no W-box sequences in their promoter regions and they were not significantly altered in expression in the transgenic GmWRKY58 and GmWRKY76 plants (Figs 9 and 10A). ChIP-qPCR assays showed that GmWRKY58 and GmWRKY76 bind to promoters of some flowering-time genes (Fig. 10B, C), suggesting their direct regulation by the two WRKY proteins. Notably, FLM, an inhibitor of flowering, and PIF4, a positive regulator of flowering, have been established to play critical roles in induction of flowering by certain environmental conditions such as elevated temperature (Lee *et al.*, 2013). Enrichment of W-box sequences in the promoters of both positive and negative regulators of plant flowering raises the possibility of a potentially complex network of regulation of flowering-time genes by WRKY proteins that could contribute to altered flowering time under stress conditions.

In soybean, *GmWRKY58* and *GmWRKY76* are differentially expressed at different stages of leaf growth. The expression of the two genes is at low levels in both early and late stages of leaf development, but they are expressed at substantially elevated levels during the stages of leaves expansion (Fig. 6). This expression pattern for GmWRKY58 and GMWRKY76 is consistent with their critical role as positive regulators of leaf growth based on the severely stunted leaves of *GmWRKY58*- and *GMWRKY76*-silenced soybean plants (Fig. 11). Thus, there appears to be a transcriptional cascade in which GmWRKY58 and GmWRKY76 are first activated in response to unknown developmental cues. Based on the highly responsive nature of both GmWRKY58 and GmWRKY76 to H_2_O_2_, cellular reactive oxygen species (ROS) or redox state, which are known to function as signals not only for stress responses but also growth and development (Ren *et al.*, 2002; Das and Maulik, 2003, 2004; Gill and Tuteja, 2010), could function as possible cellular cues for developmentally regulated expression of *GmWRKY58* and *GmWRKY76*. *GmWRKY58*- and *GmWRKY76*-silenced soybean plants were stunted not only in leaf growth but also in overall plant status due to inhibited stem growth. Soybean stem growth habit is a critical adaptation and agronomic trait (Tian *et al.*, 2010; Ping *et al.*, 2014). The termination of soybean apical stem growth leads to a determinate stem upon the onset of floral induction of the shoot apical meristem (Tian *et al.*, 2010; Ping *et al.*, 2014). Although the molecular basis for determining the soybean stem growth habit has not been fully established, recent studies have identified a number of genes that are important in its control. Importantly, some of the genes identified, including Dt1 and Dt2, which determine soybean stem growth habit, are homologous to Arabidopsis flowering-time/flower development genes (Tian *et al.*, 2010; Ping *et al.*, 2014). Therefore, potential soybean target genes of GmWRKY58 and GmWRKY76 may include those that regulate the growth or fate of the apical meristem, similar to those well-established flowering-time genes in Arabidopsis.

Despite their close similarity in protein sequences, overexpression of AtWRKY70 and AtWRKY54 does not significantly alter flowering time in transgenic plants. In addition, *atwrky70 atwrky54* mutants are normal in plant growth, morphology, and development. By contrast, overexpression of GmWRKY58 and GmWRKY76 in Arabidopsis altered flowering time (Fig. 8) but did not have major effects on plant disease resistance or abiotic stress tolerance (see Supplemental Figs 2 and 3). Thus despite their structural similarity, the biological functions of GmWRKY58 and GmWRKY76 have diverged from those of AtWRKY70 and AtWRKY54. In fact, almost all analyzed WRKY proteins recognize the TTGACC/T W-boxes *in vitro* but often have different biological functions. This is probably due to a number of important reasons. First, different WRKY proteins may have significant or subtle differences in their DNA-binding specificity and affinity, which has been demonstrated for even closely related WRKY proteins through *in vitro* assays using DNA molecules that differ not only in the TTGACC/T W-box consensus sequences but also in the flanking sequences (Brand *et al.*, 2010, 2013). Second, even for those WRKY proteins with the same binding specificity and affinity *in vitro*, their *in vivo* DNA-binding properties could differ significantly. This is because *in vitro* binding assays usually use naked DNA probes. *In vivo*, however, the chromosomal DNA is packaged around histone proteins to form nucleosomes, the fundamental repeating units of eukaryotic chromatin. As a result, a sequence-specific DNA-binding protein may not have access to its *in vivo* genomic targets if they are occupied by nucleosomes. In a previously published study, it has been shown that the NF1 transcription factor from *Caenorhabditis elegans* recognizes the TTGGCA(N)3TGCCAA consensus sequence *in vitro* (Whittle *et al.*, 2009). This sequence occurs 586 times in the genome of *C. elegans* but ChIP assays identified only 55 genomic binding sites of NF1(Whittle *et al.*, 2009). The reason why GmWRKY58 and GmWRKY76 could bind to the W-box elements of the flowering-time gene promoters could be due to their WRKY-domain-flanking sequences that directly or indirectly affect chromatin remodeling and nucleosome phasing. Third, depending on the protein sequences flanking the DNA-binding domains, different WRKY transcription factors can function as transcriptional activators or repressors and as a result can play different biological functions. Therefore, the biological function of a WRKY transcription factor is determined not only by its highly conserved WRKY DNA-binding domain but also by its other functional domains or motifs that dictate or modulate its access to genomic targets and transcriptional activity. Finally, differential expression patterns of different WRKY genes and post-translational modification of their products could also affect their biological functions. Indeed, we have recently compared Arabidopsis AtWRKY33 and AtWRKY25 for their differential roles in plant disease resistance and stress tolerance (Zhou *et al.*, 2015). Unlike AtWRKY25, AtWRKY33 plays a critical role in plant resistance to necrotrophic pathogens based on the analysis of both overexpression lines and knockout mutants (Zheng *et al.*, 2006; Lai *et al.*, 2011; Merz *et al.*, 2015). Through molecular complementation of the *atwrky33* mutants using mutated or truncated WRKY gene constructs, we have discovered that both the extended C-terminal domain and its stronger pathogen-responsive promoter are important for the critical role of AtWRKY33 in plant defense against necrotrophic pathogens (Zhou *et al.*, 2015). Likewise, Arabidopsis AtWRKY70 and AtWRKY54 share high sequence identify with soybean GmWRKY58 and GmWRKY76, not only in the conserved WRKY domains but also in the N-terminal region (Fig. 1). On the other hand, their C-terminal regions are relatively divergent (Fig. 1) and might be responsible for their distinct biological functions. Through structural and functional dissection of close WRKY homologs such as Arabidopsis AtWRKY70 and AtWRKY54 and soybean GmWRKY58 and GmWRKY76, it should be possible to identify important protein sequences and DNA/RNA elements critical for the distinct biological functions of WRKY proteins. This knowledge will lead to a better understanding of not only the complex mode of action of WRKY transcription factors but also the fundamental regulatory mechanisms of plant growth, development, and responses to environmental conditions.

## Supplementary Data

Supplementary data are available at *JXB* online.

**Figure S1.** Expression of *GmWRKY58* and *GmWRKY76* in transgenic Arabidopsis plants.

**Figure S2.** Response of transgenic *GmWRKY58*- and *GmWRKY76*-overexpressing Arabidopsis plants to *Pseudomonas syringae*.

**Figure S3.** Germination rates of transgenic *GmWRKY58*- and *GmWRKY76*-overexpressing Arabidopsis plants.

**Table S1.** Arabidopsis, soybean, and rice Group III WRKY proteins used in the phylogenetic analysis.

**Table S2.** Primers used in qRT-PCR.

**Table S3.** Primers used in ChIP-qPCR.

Supplementary Data
