# Supplementary material for: Functional analysis of structurally related soybean GmWRKY58 and GmWRKY76 in plant growth and development
Source: J Exp Bot. 2016 Jun 21;67(15):4727–42. doi: 10.1093/jxb/erw252 (PMC4973743; doi:10.1093/jxb/erw252)
Supplement: Supplementary Data [file supp_67_15_4727__index.html]

Functional analysis of structurally related soybean GmWRKY58 and GmWRKY76 in plant growth and development — Functional analysis of structurally related soybean GmWRKY58 and GmWRKY76 in plant growth and development — Supplementary Data 

# Functional analysis of structurally related soybean GmWRKY58 and GmWRKY76 in plant growth and development

## Supplementary Data

Data files

- supplementary\_figures\_S1\_S3.pdf - Supplementary Data
- supplementary\_Tables\_S1\_S3.pdf - Supplementary Data
