# Supplementary material for: Functional analysis of structurally related soybean GmWRKY58 and GmWRKY76 in plant growth and development
Source: J Exp Bot. 2016 Jun 21;67(15):4727–42. doi: 10.1093/jxb/erw252 (PMC4973743; doi:10.1093/jxb/erw252)
Supplement: Supplementary Data [file supp_erw252_supplementary_figures_S1_S3.pdf]

Supplemental Figure 1

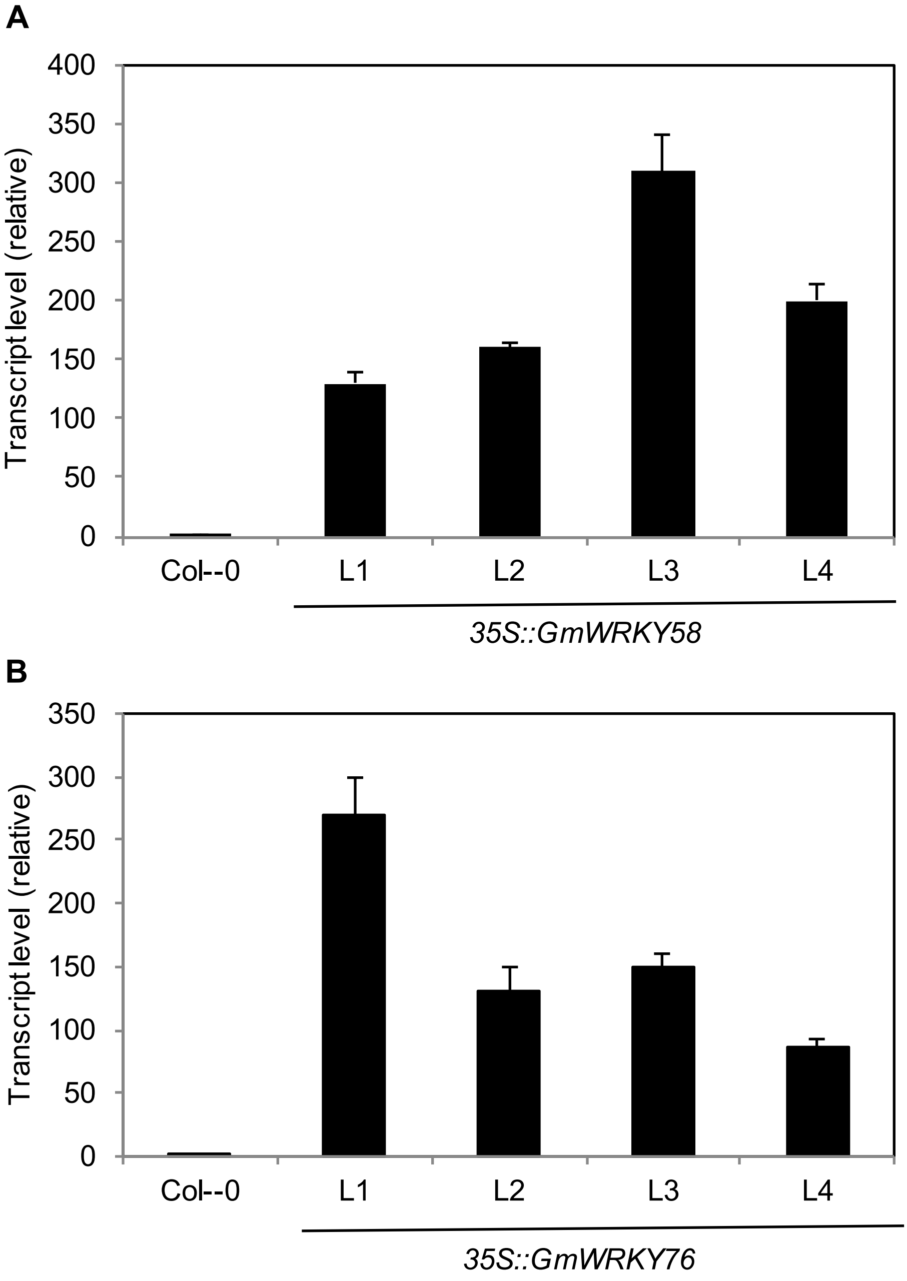

Supplemental Figure 1. Expression of GmWRKY58 and GmWRKY76 in transgenic Arabidopsis plants.

The leaf samples were collected from 3-week-old seedlings of control Col-0 and transgenic *GmWRKY58* and *GmWRKY76* plants for total RNA isolation. Expression of *GmWRKY58* and *GmWRKY76* was analyzed by qRT-PCR using an Arabidopsis actin gene as an internal control. Values represent the means and standard errors of three replicates.

Supplemental Figure 2

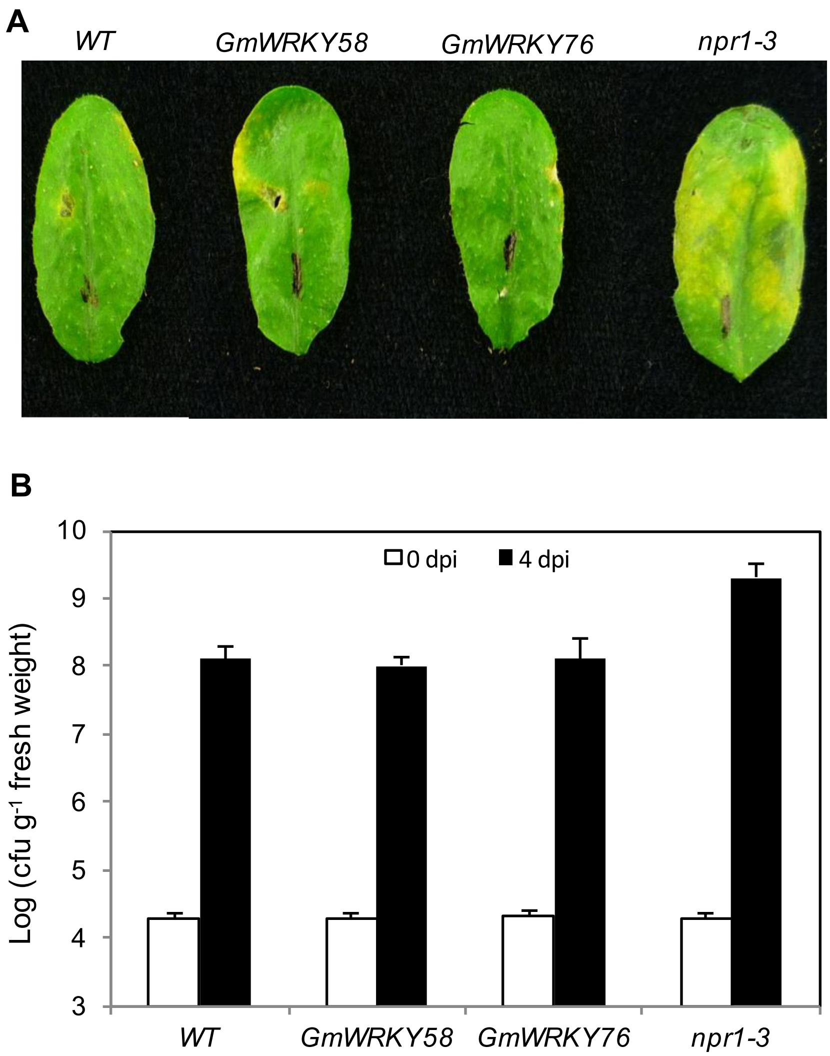

Supplemental Figure 2. Response of transgenic *GmWRKY58*- and *GmWRKY76*-overexpressing Arabidopsis plants to *P. syringae*.

(A) Disease symptom development in the control Col-0 (WT), transgenic *GmWRKY58* and *GmWRKY76* and *npr1* mutant plants. Plants were infiltrated with a suspension of PstDC3000 (OD_{600}=0.0002 in 10 mM $MgCl_2$). Pictures of representative inoculated leaves taken at 4 dpi.

(B) Bacterial growth in in the control Col-0 (WT), transgenic *GmWRKY58* and *GmWRKY76* and *npr1* mutant plants. Pathogen inoculation of plants was performed as in A. Samples were taken at 0 or 4 dpi to determine the bacterial growth. The means and standard errors were calculated from 10 plants for each mutant.

Supplemental Figure 3

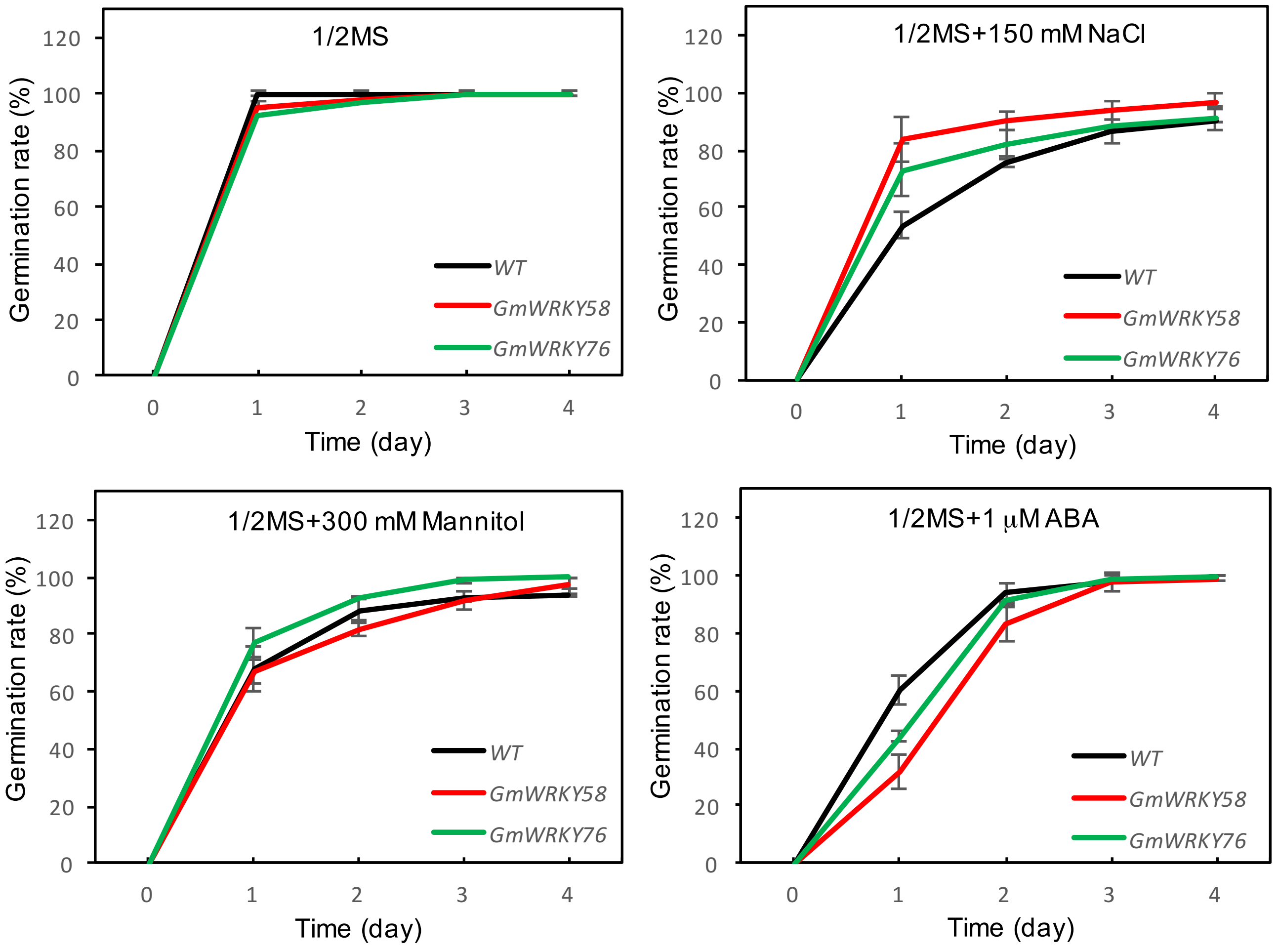

Supplemental Figure 3. Germination rates of transgenic *GmWRKY58*- and *GmWRKY76*-overexpressing Arabidopsis plants.

Seeds of control Col-0 (WT) and transgenic plants were surface-sterilized and sown on ½ MS medium containing indicated concentrations of NaCl, mannitol or ABA. The plates were incubated for 48 hours at 4°C and then transferred to an incubator at 25°C (12h day/night photoperiod). The germination rates as percentages of seeds sown were scored from approximately 100 seeds in three petri dishes at indicated days at 25°C.
