# Supplementary material for: Functional analysis of structurally related soybean GmWRKY58 and GmWRKY76 in plant growth and development
Source: J Exp Bot. 2016 Jun 21;67(15):4727–42. doi: 10.1093/jxb/erw252 (PMC4973743; doi:10.1093/jxb/erw252)
Supplement: Supplementary Data [file supp_erw252_supplementary_Tables_S1_S3.pdf]

Supplemental Table 1. Group III WRKY proteins used in the phylogenetic analysis

Gene name	Gene identifier	Gene name	Gene identifier
AtWRKY30	At5g24110	OsWRKY15	Os01g46800
AtWRKY38	At5g22570	OsWRKY18	Os01g60520
AtWRKY41	AT4G11070	OsWRKY19	Os05g49620
AtWRKY46	AT2G46400	OsWRKY20	Os01g60540
AtWRKY53	AT4G23810	OsWRKY21	Os01g60640
AtWRKY54	AT2G40750	OsWRKY22	Os01g60490
AtWRKY55	AT2G40740	OsWRKY40	Os11g02530
AtWRKY62	AT5G01900	OsWRKY41	Os11g45920
AtWRKY63	AT1G66600	OsWRKY44	Os03g21710
AtWRKY64	AT1G66560	OsWRKY45	Os05g25770
AtWRKY66	AT1G80590	OsWRKY46	Os11g02480
AtWRKY67	AT1G66550	OsWRKY47	Os07g48260
AtWRKY70	AT3G56400	OsWRKY48	Os05g40060
GmWRKY12	Gm01g43420	OsWRKY50	Os11g02540
GmWRKY20	Gm08g02580	OsWRKY52	Os11g02470
GmWRKY43	Gm03g41750	OsWRKY54	Os05g40080
GmWRKY55	Gm16g02960	OsWRKY55	Os03g20550
GmWRKY57	Gm18g44560	OsWRKY56	Os12g02440
GmWRKY58	Gm04g40130	OsWRKY64	Os12g02450
GmWRKY60	Gm07g06320	OsWRKY65	Os12g02470
GmWRKY74	Gm09g41050	OsWRKY69	Os08g29660
GmWRKY76	Gm06g14720	OsWRKY74	Os09g16510
GmWRKY145	Gm04g41701	OsWRKY75	Os05g25700
GmWRKY148	Gm13g34251	OsWRKY81	Os12g02400

GmWRKY160	Gm04g40121	OsWRKY90	Os05g14370
GmWRKY163	Gm16g34590	OsWRKY91	Os05g40070
GmWRKY165	Gm06g14731	OsWRKY93	Os06g06360.
GmWRKY166	Gm13g34261	OsWRKY95	Os12g02440
GmWRKY167	Gm13g34281	OsWRKY97	Os12g02420
GmWRKY168	Gm13g34240	OsWRKY98	Os09g09630
GmWRKY171	Gm14g36430	OsWRKY99	Os10g18099
GmWRKY172	Gm14g36446	OsWRKY104	Os11g02520
GmWRKY173	Gm14g36438	OsWRKY115	Os07g27670
GmWRKY174	Gm03g00460		

Supplemental Table 2. Primers for qRT-PCR

Gene name	Gene identifier	Primers
GmWRKY58	Gm04g223300	F: CTTGGTCCTGACGCAAATGA<br>R:TCCTTGAAATCAGACCAAAA
GmWRKY76	Gm06g142000	F: TCTGTGTTGACTTCTTCTTC<br>R: TCCTCAGAACTCGGATCATT
AP1	At1G69120	F: AACCAAGGCCACAATATGCC<br>R: CGGGTTCAAGAGTCAGTTCG
ARP6	At3g33520	F: GAGTCTGTGGTGGTGATGGA<br>R: CTGCAGGCTGGAATAACGTC
CO	At5g15840	F: GTGATAAGGATGCCAAGGAG<br>R: AGTTTAAGCGGAACAACTCTA
FLC	At5g10140	F: CTCTACAGCTTCTCCTCCGG<br>R: TCCCACAAGCTTGCTATCCA
FLM	At1g77080	F: GATAGAAGCGCTGTTCAAGC<br>R: CAGCAACGTATTCTTTCCCAT
FT	At1g65480	F: TACGAAAATCCAAGTCCCACTG<br>R: AAACTCGCGAGTGTTGAAGTTC
LEAFY	AT5G61850	F: GCCACTTGTGAACATCGCTT<br>R: AATACCGCCAACTAAAGCCG
PIF4	At2g43010	F: CAGCTTCAAGTGATGTGGATG<br>R: CATAACCGGAAATCGAGGTAA
SOC1	At2g45660	F: AAACGAGAAGCTCTCTGAAAAG<br>R: AAGAACAAGGTAACCCAATGAAC
SVP	At2g22540	F:GAAGAGAACGAGCGACTTGG<br>R:GAGCTCTCGGAGTCAACAGG
GmACTIN	Gm18g290800	F: GTGCACAATTGATGGACCAG<br>R: GCACCACCGGAGAGAAAATA
AtACTIN	At3G18780	F: CCTCAAAGACCAGCTCTTCC<br>R: TCTTTGCTCATACGGTCAGC

Supplemental Table 3. Primers for ChIP-qPCR

Gene name	Gene identifier	Primers
AP1	At1G69120	F: TCCGTCGACGCAATACATTA<br>R: CGAAACAAAATCTGAACCAACC
ARP6	At3g33520	F: ACGTTTGTTCCGATGAATCC<br>R: TGGCGTCGTTGTACTTTTTG
CO	At5g15840	F: GACTACTTGGCGGATTCGAG<br>R: TGAATGCATCTGCATCATTT
FLC	At5g10140	F: CACTCTCGTTTACCCCCAAA<br>R: AGGGCTTTGTGCCCTAATTT
FLM	At1g77080	F: ACGGCAGAACCTACGACCTA<br>R: CATCTTGTCTTGATGCATTGG
FT	At1g65480	F: GTGGCTACCAAGTGGGAGAT<br>R: TAACTCGGGTCGGTGAAATC
LEAFY	AT5G61850	F: CAATTTCCCAGCAAGACACA<br>R: TGACCACTCGAGCAACTTCT
PIF4	At2g43010	F: TGACAAAAACACCCTCAAATCA<br>R: TCTGGGACATTGTGTTGGAA
SOC1	At2g45660	F: TGGGTTTGGTTTCATTTGGT<br>R: GGGTGGGAGAAGACTGATGA
SVP	At2g22540	F: TACAAACCAAGGCCGAACTC<br>R: CCTGAAGAACATCAAGGGCTA
